# Digital quantification of soft tissue volumetric changes after scaling and root planning

**DOI:** 10.4317/medoral.26766

**Published:** 2024-08-18

**Authors:** Irene Vallejos-Juárez, Antonio Fons-Font, Rubén Agustín-Panadero, Eva González-Angulo, Jorge Alonso-Pérez-Barquero, Carla Fons-Badal

**Affiliations:** 1 Dentist, private practice, Spain; 2 Professor, Department of Oral Medicine, Faculty of Medicine and Dentistry, University of Valencia, Spain; 3 Associate Professor, Department of Oral Medicine, Faculty of Medicine and Dentistry, University of Valencia, Spain

## Abstract

**Background:**

Monitoring the outcome and evolution of periodontitis treatment requires analyzing changes in the periodontium. However, traditional methods for analyzing volumetric changes in periodontal soft tissues have limitations due to their invasiveness or inaccuracy. The aim of this study was to measure the volumetric changes in periodontal tissues following scaling and root planing treatment using digital methods, such as the superimposition of pre- and post-treatment STL files

**Material and Methods:**

The study started with an initial periodontal examination and intraoral scanning. Periodontal treatment was then performed, and at the one-month re-evaluation, the same records were repeated. Finally, the clinical data and STL files of pre- and post-treatment scans were compared.

**Results:**

in terms of clinical data, there was a mean decrease in probing depth of 0.34 ± 0.54 mm and a significant decrease in bleeding rate. Digital measurements showed a mean loss in height of 0.196 ± 0.188 mm and width of 0.344 ± 0.338 mm.

**Conclusions:**

Quantifying periodontal tissue changes after scaling and root planing was possible by superimposing STL files. Post-treatment gingival tissue shrinkage occurred in both height and width, which was not visible with conventional recordings.

** Key words:**Scaling and root planing, recession, intraoral scan, periodontal volumetric changes, periodontal treatment.

## Introduction

Periodontitis is a multifactorial disease that requires treatment involving the removal of plaque and calculus deposits through scaling and root planing (1-5). This procedure produces changes in the supporting tissues to achieve gingival health, including cementum removal at the tooth level, and gingival recession or decreased probing depth in the surrounding soft tissues (6-9). One of the main concerns for patients undergoing periodontal treatment is the dimensional changes of their soft tissues. Tissue retraction can be difficult to monitor objectively and is often perceived as unsightly. This can lead to the patient's subjective perception dominating the situation.

Several methods are available to quantify volumetric changes, including transmucosal probing, Cone-Beam Computed Tomography, and ultrasonic determination. However, these methodologies have limitations. Some are invasive, while others are not very accurate and, therefore, difficult to apply on a daily basis (10).

The use of intraoral scanners in dental practice offers new opportunities for monitoring and follow-up of treatments. Computer-assisted image analysis is a viable option for evaluating gingival tissues in periodontology (10-13). The ability to superimpose 3D models at various spatial points allows for non-invasive evaluation of soft tissue dynamics and visualization of gingival thickness, along with its stability over time (14).

Therefore, the aim of this study was to quantify the volumetric changes in periodontal tissues after scaling and root planing treatment using digital methods by superimposing pre- and post-treatment STL files. The results obtained were then compared with those obtained using traditional evaluation systems.

## Material and Methods

The study was conducted for one year at the Prosthodontics and Occlusion Teaching Unit of the Department of Stomatology at the University of Valencia. Twelve patients already scheduled for scaling and root planing to treat their periodontal pathology were selected. The inclusion criteria comprised patients with active periodontal disease who were of legal age and able to sign the informed consent form. Patients were excluded if they had undergone dental cleaning within the last six months, taken antibiotics or anti-inflammatory drugs within the previous three months, or were taking medication that could affect the anatomy of their gums, such as phenytoin, cyclosporine, or nifedipine.

The study received approval from the University of Valencia's Ethics Committee for Human Research under number 1936800 and verification code K5L1O205CI0J4MCB.

- Clinical records and treatment

The study participants underwent a periodontal examination, which included a full-mouth pocket depth and recession probing (PST0) with a Williams Hu-Friedy periodontal probe (QulixR-PW, Chicago, USA), a dichotomous bleeding index, a full-mouth radiographic series (XCP-RINN® film holder), and an intraoral scan with the TRIOS 3 scanner (3Shape®, Copenhagen, Denmark) representing the patient's baseline (STL0). Subsequently, the patients received periodontal treatment, which included ultrasonic scaling (SP Newtron, Satelec Acteon, Olliergues, France) and scaling and root planing with Hu-Friedy Gracey curettes (Chicago, IL, USA). The treatment was performed in quadrants, and all quadrants were instrumented during the final session to ensure that all teeth had been instrumented at the same time for re-evaluation. Additionally, the patients were also given oral hygiene instructions. At the periodontal re-evaluation, which occurred one month after the end of treatment, the clinical record and intraoral scan were repeated to assess the treatment response and any changes that occurred in the periodontal tissues. The second clinical record (PST1) and intraoral scan (STL1) were used for this purpose. Radiographic examination was not repeated as bone changes could not be evaluated within this time interval.

- Digital protocol

**Figure 1:**
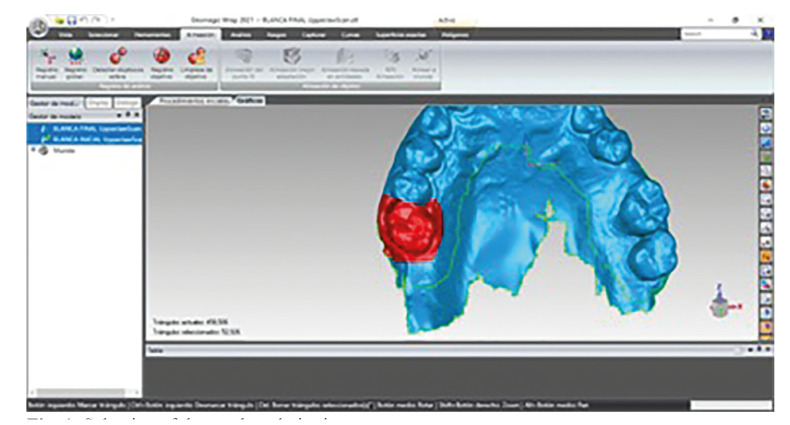
Selection of the tooth and gingiva.

STL files 0 and 1 were imported into the Geomagic Wrap 2021 software (Geomagic Verify medTM, Geomagic, Morrisville, USA). To reduce errors during alignment of the files, a segmentation of each of the teeth to be evaluated was performed, selecting the corresponding portion of the gingiva (Fig. 1).

**Figure 2:**
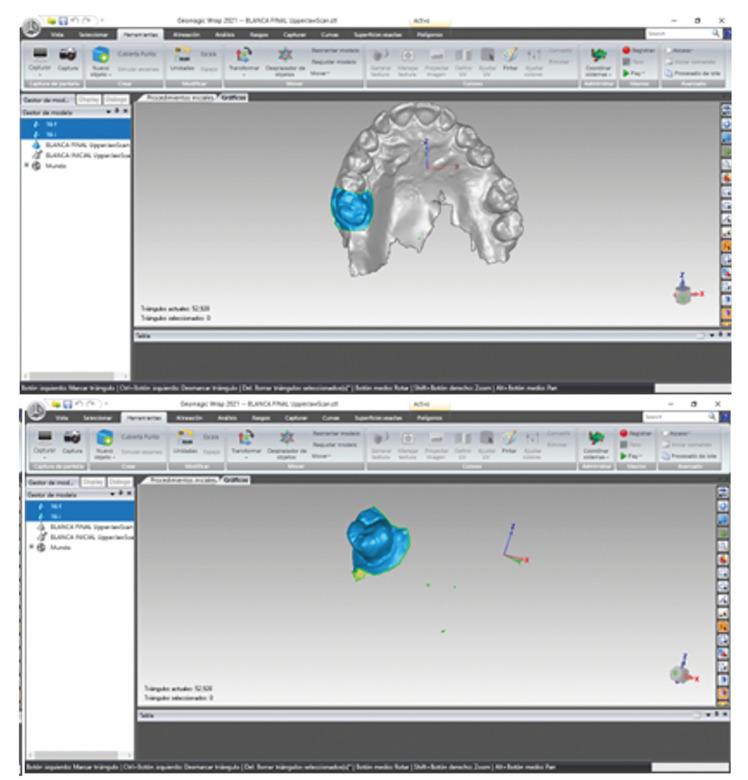
New file STL0 is created with the information of teeth and gingiva.

A new STL file was obtained for each of the teeth in the study STL0 (Fig. 2). After segmentation, the alignment was performed individually on each tooth STL0 and STL1 using the best fit algorithm. Only the clinical crown was selected, since its surface had not been modified between the initial (STL0) and final (STL1) situation. Once aligned, the files were then imported into GOM Inspect 2018 software (GOM GmbH, Braunschweig, Germany) for measurements.

**Figure 3:**
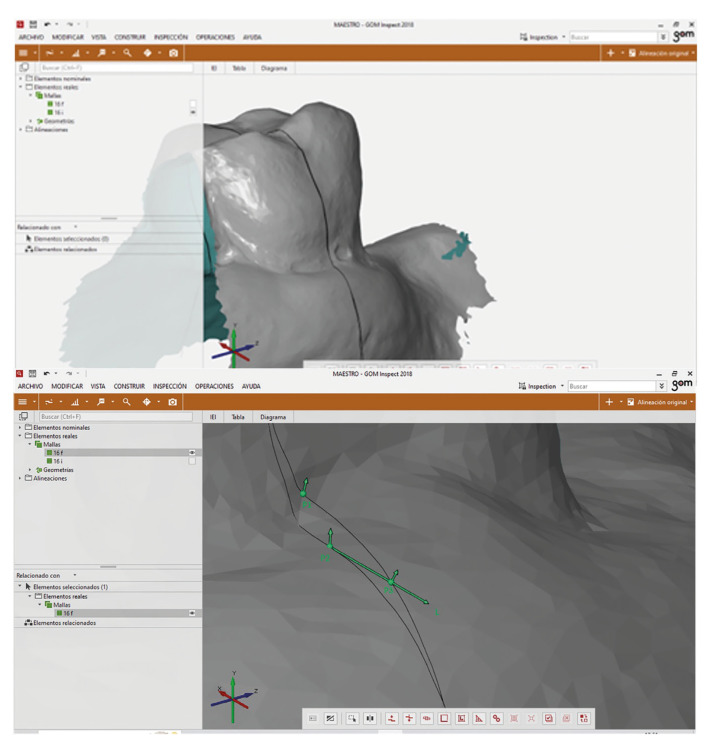
A section through de longitudinal axis was created in the STL0 and STL1 and a screenshot from GOM Inspect software, where the intersection point (P3) between the created line (L) and the initial mesh (STL0) can be seen.

First, a section was made on each pair of files (STL0 and STL1) following the longitudinal axis (Fig. 3). Over these two sections 3 surface points were created. Point 1 (P1) corresponds to the most coronal point of the gingival sulcus on the STL0 file and point 2 (P2) corresponds to the most coronal point of the gingival sulcus on the STL1 file. To determine point 3 (P3), a perpendicular line to the axis of the tooth (L) was drawn starting from P2. Point 3 (P3) corresponds to the point that results from the cut between the line L and the section of the STL0 (Fig. 3). Therefore, the distance between P2 and P3 represents the change in soft tissues in the horizontal direction, while the distance between P1 and P2 represents the change in soft tissues in the vertical direction.

- Statistical analysis

The data were analyzed using SPSF software with a parametric approach due to the sample size. An inferential analysis was also conducted, estimating a linear model with generalized estimating equations (GEE) to compare significant changes. Confidence intervals at 95% were provided based on the Wald chi-squared statistic, and a significance level of 5% (α=0.05) was used in the analyses.

## Results

The study analyzed 131 teeth from 12 patients, consisting of 6 females (50%) and 6 males (50%) with a mean age of 55.25 ± 10 years, ranging from 33 to 72 years old.

Following treatment, the patients' periodontal situation improved, with a decrease in probing pocket depth and bleeding rate. The initial and final periodontal charts were analyzed, considering probing pocket depth, recession, and bleeding.

A statistically significant decrease (*p*<0.001) was observed in probing pocket depth, with the average measurement reducing from 2.74 ± 0.69 mm (PST0) to 2.40 ± 0.52 mm at the reevaluation (PST1). The mean decrease was -0.34 ± 0.54 mm (95% CI: -0.51 -0.17) (Table 1).

Regarding recession, only four teeth (3.1%) had a recession of 1 mm according to the periodontal chart.

As for bleeding on probing, 74% of the teeth initially showed bleeding, which was reduced to 35.1% after treatment. Out of the total of 131 teeth, bleeding disappeared in 52 (39.7%), while only one (0.8%) showed bleeding that appeared after the treatment. The difference was statistically significant (*p*<0.001), indicating that the treatment effectively reduced the bleeding rate.

**Figure 4:**
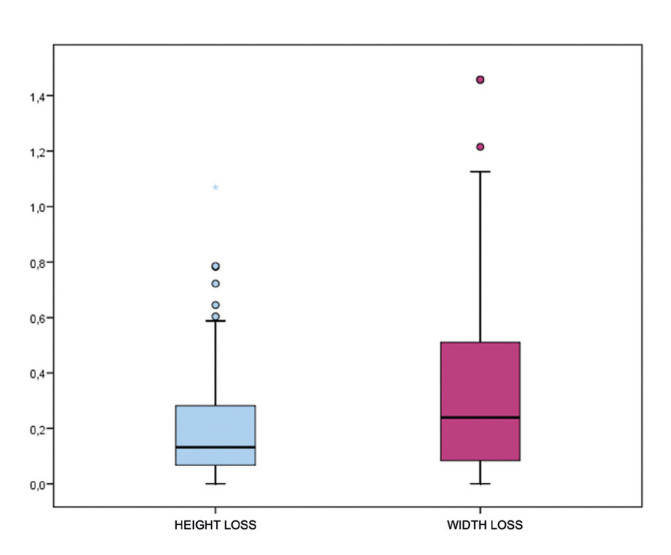
Box plot representing height loss and width loss distributions.

The comparison and digital analysis of the pre- and post-treatment STL files revealed changes in the soft tissues that were not detecTable by classical methods, such as the periodontal chart. Specifically, there was a reduction in height from 2.74 ± 0.69 mm to 2.40 ± 0.52 mm upon re-evaluation, indicating a mean height loss of 0.196 mm with a standard deviation of 0.188 mm (95% CI: 0.142-0.250). The mean loss in width was 0.344 mm with a standard deviation of 0.338 mm (95% CI: 0.219-0.469) (Table 2, Fig. 4). Both decreases were statistically significant (*p*<0.001), although the decrease in height was clinically not relevant.

## Discussion

Traditionally, gingival thickness has been determined by transmucosal probing under local anesthesia using a periodontal probe or a file with a rubber stop. However, this method can lead to an erroneous increase in volume due to rounding of the measurement and discomfort for the patient. As a result, it is not performed regularly in the clinic. These measurements are not very accurate, allow only one point to be measured to avoid excessive tissue damage, and are impractical due to patient discomfort. Therefore, alternative methods for measuring gingival thickness have been evaluated, and the digital method has been found to be a reliable alternative (15).

Several studies that have used digital techniques to measure periodontal soft tissue. However, each study has followed a different methodology and objective. Galarraga-Vinueza *et al*. in 2020 performed a study on 3D models in patients with advanced periodontitis. They analyzed the occurrence of gingival recession after combined surgical periodontal therapy using GOM Inspect software. The authors concluded that further research is necessary, particularly in regard to potential correlations between volumetric changes and recession depth measurements (16). Strebel *et al*. in 2009 evaluated volumetric changes in interdental papillae using Swissmeda software and optical impressions with scanning spray (17). Using Geomagic Wrap software, Lehmann *et al*. in 2012 calculated the volume of gingival tissue lost due to recession by digital scanning and subsequent analysis on digital models with respect to a CAD reference model using markings representing the four classes of gingival recession in Miller's classification (18). Schneider *et al*. in 2014 measured gingival recession and papilla height in 30 locations using three different methods: periodontal probe, caliper, and digital technology. They compared the measurements obtained with Geomagic Wrap software and stated that the use of digital technology improved reproducibility (19). In 2021, Hawra Al-Qallaf *et al*. performed a study on cadaver jaws to evaluate periodontal tissue. The study involved bone probing, DICOM files, and STL files. The researchers concluded that DICOM+STL file registration is a suiTable method for measuring gingival thickness in clinical and research settings of soft tissue dimensional changes without traumatizing soft tissues (20). In 2022, Fons-Badal *et al*. performed a study to measure the volume gain following connective tissue graft surgery. They used STL file comparison through Geomagic Wrap and GOM Inspect, which is similar to the method used in the present study. However, they evaluated gingival tissue gain after graft tunnel surgery (17). The main limitation of the method used in our study is the challenge of accurately selecting a measurement point. A small variation in the location of the measurement point can result in different values. One potential solution to this drawback is to evaluate the volume of the zone, which could be a promising avenue for future research.

Thanks to the comparison between initial and final STL files, the study found that there were dimensional changes at the gingival level that were not detecTable using the periodontal chart. This is because the periodontal probe used has a scale that only measures in millimeters, and therefore cannot quantify the smallest values. Specifically, there was a mean decrease in height of 0.196 ± 0.188 mm and in width of 0.344 ± 0.338 mm. There was only a slight decrease in gingival height, which is a positive outcome at the clinical level since tissue retraction is often viewed negatively by patients.

The digital method is presented as an objective technique that enables precise visual monitoring and easy storage. Additionally, it offers advantages over the conventional method by allowing for a more accurate diagnosis of the patient's oral condition, early identification of lesions, and subsequent monitoring. The possibility of observing the dimensional differences in oral tissues and the documented and objective evidence it provides can be useful in legal proceedings.

## Conclusions

1. Digital evaluation by superimposing STL files allowed for the quantification of periodontal tissue changes after scaling and root planing treatment. Gingival tissues showed a decrease in height and thickness post-treatment.

2. These changes were not appreciated with conventional recordings and, therefore, could not be quantified. Digital measurements have made it possible to quantify changes of less than 1 mm that were previously not assessable with the periodontal probe.

3. The measurement method studied represents an improvement over conventional techniques because it is non-invasive and allows for the correction of errors resulting from imprecise instruments. Further research in this area would be of great interest to address the encountered difficulties and to continue improving the standardization of the technique.

## Figures and Tables

**Table 1:** Variations in probing pocket depth according to the periodontal chart.

Clinical record	Measure	Value
PST0	N	131
	Average	2.74
	Standard deviation	0.69
	Minimum	1.50
	Maximum	5.33
	25th percentile	2.167
	Median	2.67
	75th percentile	3.17
PST1	N	131
	Average	2.40
	Standard deviation	0.52
	Minimum	1.50
	Maximum	3.83
	25th percentile	2.000
	Median	2.33
	75th percentile	2.83
DIF.PS.T1-T0	N	131
	Average	-0.34
	Standard deviation	0.54
	Minimum	-2.00
	Maximum	1.67
	25th percentile	-0.667
	Median	-0.17
	75th percentile	0.00

**Table 2:** Variation of registered height and width.

Periodontal tissue changes	Measure	Value
Height loss	N	131
	Average	0.196
	Standard deviation	0.188
	Minimum	0.000
	Maximum	1.070
	25th percentile	0.067
	Median	0.132
	75th percentile	0.283
Width loss	N	131
	Average	0.344
	Standard deviation	0.338
	Minimum	0.000
	Maximum	1.458
	25th percentile	0.082
	Median	0.240
	75th percentile	0.525
